# Inhibition of miR-let-7i Induces DC Immature Cells and Improves Skin Graft Tolerance

**DOI:** 10.1155/2022/8605621

**Published:** 2022-06-15

**Authors:** Zhibin Zheng, Yurou Yang, Hai Liu, Mingdao Hu, Peng Chen

**Affiliations:** ^1^Hepatopancreatobiliary Surgery Department, Kunming Medical University, 650101 Kunming, China; ^2^Rheumatology and Immunology Department, Affiliated Hospital of Southwest Medical University, 646000 Luzhou, China; ^3^Hepatopancreatobiliary Surgery Department 1, The Second Affiliated Hospital of Kunming Medical University, 650101 Kunming, China

## Abstract

Dendritic cells (DC) initiate the immune response in the body. They can stimulate T cell activation, proliferation, and differentiation and ultimately participate in the immune response and the immune tolerance response. The purpose of this study was to coculture DCs and T cells and subcutaneously inject DCs transfected with miR-let-7i into rhesus monkey transplantations to verify the role of miR-let-7i in allograft immune tolerance. In vitro studies found that the expression of miR-let-7i was upregulated after inducing the maturation of DCs. The low expression of miR-let-7i inhibited the maturation of DCs, promoted the differentiation of T cells into T helper T cells 2 (Th2), and inhibited T helper T cell 1- (Th1-) driven rejection. In vivo studies also obtained similar results, and subcutaneous injection of DCs transfected with miR-let-7i inhibitor prolonged the survival time of allogeneic skin transplantation. Therefore, we conclude that inhibition of miR-let-7i inhibits DC maturation and improves the tolerance of grafted skin.

## 1. Introduction

Allogeneic skin transplantation can be used to treat skin wounds and burns caused by accidents. However, immune rejection is a dangerous potential complication and the biggest risk factor for chronic rejection and dysfunction of allograft transplantation.

Skin allografts can be used to treat skin wounds and burns caused by accidents [1]. However, immune rejection after transplantation is the main cause of organ transplant failure [2, 3]. First, although immunosuppressive drugs can prolong the time of allogeneic transplantation, immunosuppressive agents cannot completely solve the rejection reaction. Second, the application of immunosuppressive agents brings the risk of cancer and infection and may produce other adverse reactions [4]. Therefore, clarifying the mechanism of immune tolerance and establishing tolerance to allografts are of great significance to avoid allograft rejection, reduce the risk of transplantation, and increase the success rate of transplantation.

MicroRNAs (miRNAs), noncoding RNA molecules with a length of 20-24 nucleotides, are considered biomarkers of transplant rejection or transplant dysfunction [5, 6]. miRNAs are key regulators of the mammalian immune system, affecting a variety of immune processes, including innate immunity, inflammation, adaptive immunity, and infection [7]. Dendritic cells (DCs) initiate the immune response. They participate in the process of capturing, processing, and presenting antigens. After contacting T cells, they stimulate T cell activation, proliferation, and differentiation and ultimately participate in the immune response and immune tolerance. In addition, DCs play a crucial role in coordinating the balance between the immune response and immune tolerance [8]. According to the degree of differentiation of DCs, DCs are divided into mature DCs (mDCs) and immature DCs (imDCs) [9]. ImDCs usually show high endocytosis capacity and low T cell activation potential, thereby inducing immune tolerance. After the antigen is captured, DCs process the selected exogenous polypeptides, transport them to the surface, and convert them into mDCs, which can induce an antidonor immune response [10, 11]. Therefore, maintaining the immature state of DCs is essential for inducing and maintaining immune tolerance. Lipopolysaccharide (LPS) is the main component of the cell walls of gram-negative bacteria, which can induce DC maturation and trigger specific T cell immunity [10, 12].

Many studies have shown that miRNAs regulate DC maturation and T cell activation [13]. The miR-let-7 cluster was one of the first miRNA families discovered, and it has been shown to be involved in the immune response process. For example, miR-let-7i is involved in regulating the immune response. Studies have shown that inhibiting miR-let-7i can regulate the Toll-like receptor pathway by targeting suppressor of cytokine signal transduction 1 (SOCS1) and reduce the dendritic cells stimulated by lipopolysaccharide (LPS) to maturity [14]. Tumor-derived exosomes (TEX) are modified by electroporation to overexpress miR-155, miR-142, and miR-let-7i, which can change the original immunosuppressive effect of TEX and induce DC maturation [15]. Compared with the untreated group, mice intravenously injected with DCs transduced with CD40 siRNA showed prolonged graft survival, increased Treg cells, fewer CD4+ and CD8+ T cells, and promoted T cell differentiation into Th2 cells [16].

The above studies indicate that miR-7i has a potential target in allograft immune tolerance, but the role of let-7i in skin graft immune tolerance remains to be explored. Therefore, this study was aimed at verifying the role of miR-let-7i in allograft immune tolerance through cell coculture and subcutaneous injection of miR-let-7i-induced DCs in rhesus monkeys.

## 2. Materials and Methods

### 2.1. Animals

Rhesus monkeys were purchased from the Experimental Animal Center of Kunming Institute of Zoology, Chinese Academy of Sciences (production license number: SCXK (Dian) K2017-0003; quality certificate: yes; and breeding method: long-term breeding in Huahongdong Park, Kunming Institute of Zoology, Chinese Academy of Sciences). The monkey farm is specially administered by the animal center, and the animals are raised in single cages. The raising method is conventional. The animal experiments were approved by the ethics committee following review by the Institutional Animal Care and Use Committee of Kunming Institute of Zoology, Chinese Academy of Sciences (IACUC number: IACUC-PE-2021-09-002). The animal outcome was rehabilitation treatment, as the animal experiment was a small survival operation and did not lead to the death of experimental animals.

### 2.2. Extraction and Culture of iDCs from Bone Marrow

After ketamine anesthesia, the rhesus monkey was fixed in the prone position on an animal experimental operating table, the posterior iliac crest was taken as a puncture point, the puncture point was routinely disinfected with iodophor three times, the puncture was performed with a 16-gauge bone needle, the occipital core was removed, and 20 ml of bone marrow was drawn with a syringe, with 10 ml drawn at a time. For separation of marrow, 10 ml of bone marrow blood was transferred into a 50 ml centrifuge tube, an equal volume of PSB was added and mixed well, an equal volume of monkey lymphocyte separation solution was transferred into a new 50 ml centrifuge tube, and the bone marrow blood was mixed with PBS at a volume of 5 ml or 10 ml. The liquid tube was slowly added to the surface of the monkey lymphocyte separation liquid, and the mononuclear cells in the bone marrow were separated according to the density gradient centrifugation method and transferred to a new centrifuge tube for use. CD34+ cells were collected by the immunomagnetic bead method, and 200 *μ*l of 1640 complete medium (Thermo Fisher Technology Co., Ltd., Shanghai, China) containing 10% FBS was added to resuspend the cells with the isolated CD34+ cells. A cell counting plate was seeded at a density of 1 × 10^6^ cells/1.5 ml cells per well into a 12-well plate. RPMI 1640 complete culture medium containing 10% FBS and the cytokines granulocyte-macrophage colony-stimulating factor (GM-CSF) (Poptec Biotechnology Co., Ltd., Suzhou, China) and IL-4 (STEMCELL Technologies Co., Ltd., Canada) (concentrations of 0.1 mg/l and 0.05 mg/l, respectively) was added to a total volume of 1.5 ml. The cells were cultured in an incubator with a volume fraction of 5% CO_2_ at 37°C. The medium was changed every two days, and CD34+ cells were induced to differentiate into immature dendritic cells on the 5th day of culture. On the 6th day of culture, the cells were collected to observe their ultrastructure by electron microscopy, and the cellular immune phenotype was identified by flow cytometry. After the 7th day of culture, the constructed lentivirus was added to transfect the immature dendritic cells; after the 9th day, the immature dendritic cells were induced to maturity.

### 2.3. Observation of Rhesus Monkey Bone Marrow Dendritic Cells

The DC suspension of each group was washed twice with 0.1 mol/l PBS, and the cell pellet was collected by low-speed centrifugation. The cells were resuspended with PBS, and the cell suspension was dropped onto a polylysine-coated coverslip and allowed to stand in a 47°C, saturated humidity incubator for 15 minutes to allow the cells to naturally settle on the coverslip. The cells were washed gently with PBS 3 times and fixed with 3% glutaraldehyde (pH 7.2~7.4) at 4°C for 1 hour. The cells were rinsed twice with PBS for 30 minutes each time and fixed with 1% osmium acid for 1 hour. The samples were rinsed twice with PBS, and then, different concentrations of alcohol were used to dehydrate the samples step by step. The preparation of the ring, dry coating, etc. was completed by the electron microscope room. The morphological characteristics of the cells were observed.

### 2.4. Phenotypic Identification of Rhesus Monkey Bone Marrow Dendritic Cells by Flow Cytometry

The DC suspensions of each group were collected and centrifuged at 800 rpm for 10 min at room temperature. The supernatant was discarded, and the cells were washed twice with 0.01 mol/l PBS. The cell concentration was adjusted to 5 × 10^5^/ml. Then, 0.5 *μ*l each of PE-labeled monoclonal antibodies CD80, CD83, and MHC-II (Abcam, Cambridge, MA, USA) and the isotype control were added, mixed well, and incubated in a refrigerator at 4°C in the dark for 30 min. The cells were washed twice with PBS, transferred to a flow cytometer, and subjected to a light-shielding operation. Then, detection was performed by a flow cytometer (Thermo Fisher Technology Co., Ltd., Shanghai, China).

### 2.5. MLR Mixed Reaction System

T cells were extracted from 15 ml of peripheral blood from rhesus monkeys by the nylon wool column method. After counting the cells, 5 × 10^5^ cells were inoculated per well in a 12-well plate, and IL-2 and RPMI 1640 medium containing 10% FBS were added for complete culture. The total volume was brought to 1.5 ml. T cells (5 × 10^5^) were mixed with the abovementioned transfected imDCs in a 96-well culture plate at a ratio of 5 : 1, with 2 replicate wells prepared for each group. Each well had a total volume of 200 *μ*l of culture medium. The stimulated cells were treated according to different methods. The stimulated cells were divided into the DC, LPS+DC, imDC, OE/si-miR-let-7i-transfected imDC, LPS+imDC, and LPS+OE/si-miR-let-7i-transfected imDC groups. The time required to stimulate cell culture in the DC, LPS+DC, LPS+imDC, and transfected imDC groups was 9 days; the stimulation cell culture time of the imDC group was 7 days; and the stimulation cell culture time of the group was 8 days.

### 2.6. CCK-8 Detection

T cells (5 × 10^5^) were mixed with the abovementioned transfected imDCs in a 96-well culture plate at a ratio of 5 : 1. Each group had 2 replicate wells, and each well had a total volume of 200 *μ*l of culture medium. After culturing for 3 days, l *μ*g/ml OVA323-339 was added to each well. After 24 hours of incubation, the level of imDC response to T cell proliferation was detected by the CCK-8 method. The specific steps were as follows: the suspended T cells in each group were collected after 24 hours of cell treatment, the suspension was mixed, and 100 *μ*l was inoculated into a 96-well plate. Two replicate wells were prepared for each group, along with a cell-free blank control group. The samples were incubated in a 37°C, 5% CO_2_ incubator. Ten microliters of CCK-8 solution was added to each well at 0, 24, 48, 72, and 96 h and incubated for 1 h. Then, the absorbance value of each well was measured at 450 nm [17] on a Synergy HT microplate reader (Boten Instrument Co., Ltd, Vermont, USA).

### 2.7. Quantitative RT–PCR

In short, total RNA was collected from cells, reverse transcribed into cDNA, and amplified using SYBR premix ex Taq II (Takara, Dalian, China). Reverse transcription reactions were incubated at 16°C for 30 min, 42°C for 42 min, and 85°C for 5 min. The PCR protocol consisted of 40 cycles of 95°C for 10 s and 60°C for 1 min, followed by a heat denaturation protocol. U6 was used as an endogenous control. The 2^-∆∆Ct^ method was used for calculation.

### 2.8. Transfection of let-7i Mimics and Inhibitors

let-7i mimics or inhibitors (60 nm; Gene Pharma, Shanghai, China) were transfected into DCs according to the instructions provided for Lipofectamine 2000 (Invitrogen, Carlsbad, CA) and were used for experiments 12 h after transfection.

### 2.9. Western Blot

RIPA lysis buffer (Thermo Fisher Technology Co., Ltd., Shanghai, China) was used to extract total protein from tissues and cells. The protein concentration was determined by a bicinchoninic acid (BCA) kit (Beyotime Biotechnology Co., Shanghai, China). A polyacrylamide gel of the corresponding concentration was prepared according to the molecular weights of the proteins, the sample amount was adjusted according to the protein concentration, and electrophoresis was performed. After the electrophoresis was completed, the proteins were transferred to the PVDF membrane pretreated with formaldehyde (transfer conditions were 4°C, 90 mA, 15 h) and blocked with 5% skimmed milk powder or BSA for 1 h, the diluted primary antibodies IL-2, IL-4, IL-10, and IFN-*γ* (all from Abcam, Cambridge, MA, USA) were added, and the blots were incubated at room temperature for 2 h. Then, secondary antibodies were added and incubated at room temperature for 1 h. The bands were imaged using ECL color development, and a gel imaging system was used for imaging analysis. For semiquantitative determination of expression levels, ImageJ was used for the analysis of band gray values.

### 2.10. Animal Surgery

The experiment was allogeneic transplantation. Two rhesus monkeys with the same O blood type and Rh factor results were randomly divided into donor and recipient, and each of them was used as a donor and a recipient. The basic information and group classification of experimental animals are shown in Tables 1 and 2. Three places were selected on the back of each monkey as the donor and recipient, and skin grafts were taken from the site. On the day before transplantation, the 2nd day after the operation, the 4th day after the operation, and the 6th day after the operation, the cells were induced, cultured, transfected, and collected in the laboratory in advance by subcutaneous injection at the transplantation site, where X-NC represented immature dendritic cells transfected with empty vector gene by virus derived from peripheral blood of X monkeys and was estimated to be approximately 1 × 10^7^/kg, approximately 0.2 ml; X-inhi represented X monkey peripheral blood-induced and cultured virus-transfected immature dendritic cells that inhibit the miRNA-let-7i gene. According to the reference calculation, it was approximately 1 × 10^7^/kg or approximately 0.2 ml; X-PBS was provided at the same dose of PBS solution, approximately 0.2 ml. On the 5th day after transplantation, 1/2 of the skin grafts of each transplantation site were removed, and the remaining 1/2 of the skin grafts were removed on the 10th day after the operation for testing.

### 2.11. ELISA Method to Detect the Expression of Inflammatory Factors

The contents of IL-2, IL-4, IL-10, and INF-*γ* in the cell supernatant were detected according to the instructions of the ELISA kit. A standard curve was drawn through the standard, and then, the contents of IL-2, IL-4, IL-10, and INF-*γ* in the sample were calculated according to the OD value of the sample.

### 2.12. HE Staining

Transparent agents I and II were used for 15 minutes each, and the samples were dewaxed in gradient ethanol to water and rinsed with tap water, and the nuclei were stained with hematoxylin for 5 minutes. The samples were then rinsed with tap water until the color turned purple. Separation was performed with 1% hydrochloric acid ethanol for 2 s to remove cytoplasmic staining, and tap water was used to rinse the samples until the color turned blue. The samples were then stained with eosin for 2 min at room temperature, dehydrated with an ethanol gradient, treated with transparent agents I and II for 15 min each until transparent, and sealed with neutral gum. The samples were then observed and imaged under an optical microscope.

### 2.13. Statistical Analysis

All data are expressed as the mean ± SD. All experiments were repeated at least 3 times. Student's *t* test was used to compare the two groups. One-way ANOVA was used to compare multiple groups. GraphPad Prism 8.0 (GraphPad Software Inc., San Diego, CA, USA) was used for analysis. *P* < 0.05 was considered a statistically significant difference.

## 3. Results

### 3.1. In LPS-Induced DC Mature Cells, the Expression of miR-let-7i Is Increased

**Figure 1:**
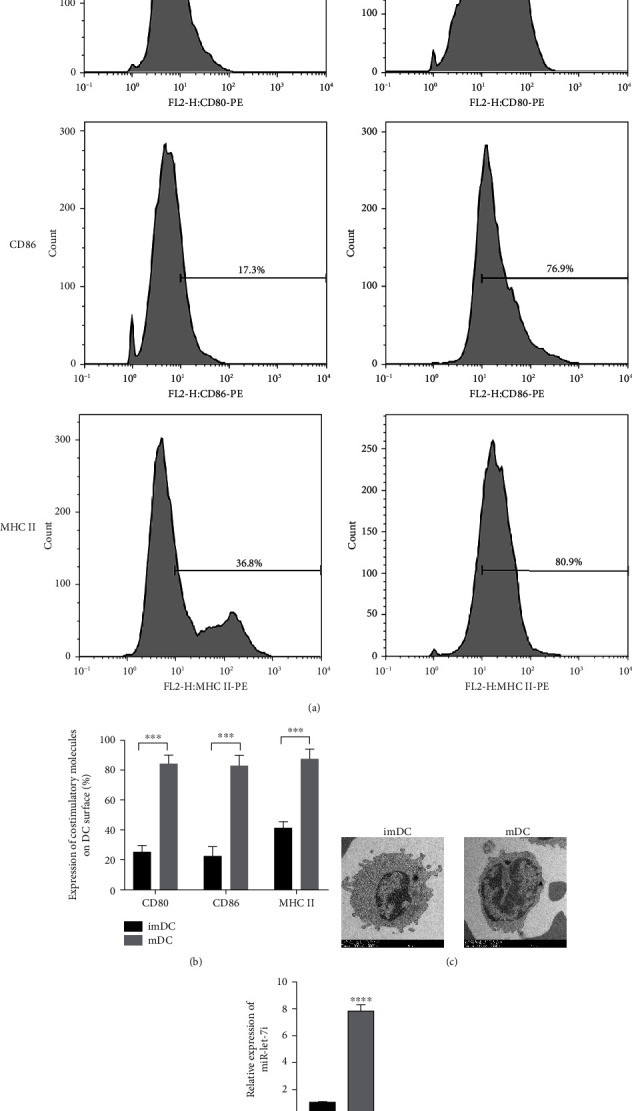
LPS induces imDC maturation. (a and b) Flow cytometry sorting for CD80, CD86, and MHC II. (c) Transmission electron microscopy to observe the morphological differences of imDCs and mDCs. (d) RT–qPCR to detect the expression of miR-let-7i in each group. Each experiment was repeated at least 3 times, ^∗^*P* < 0.05, ^∗∗^*P* < 0.01, ^∗∗∗^*P* < 0.001, and ^∗∗∗∗^*P* < 0.0001.

Compared with immature imDCs induced by LPS, the results of flow cytometry showed that the surface markers CD80, CD86, and MHC II of mature dendritic cells (mDCs) induced by LPS increased (Figures 1(a) and 1(b)). Transmission electron microscopy revealed the morphological differences between imDCs and mDCs. The results showed that the imDCs had a smooth surface with few burr protrusions. On the surface of mDCs, there are many branch-like protrusions with different lengths and shapes (Figure 1(c)). RT–qPCR results showed that the expression of miR-let-7i in mDCs increased (Figure 1(d)). The above results indicate that LPS can induce imDCs to maturity and promote the expression of miR-let-7i.

### 3.2. LPS Induces DC Maturation and Increases the T Cell Response

**Figure 2:**
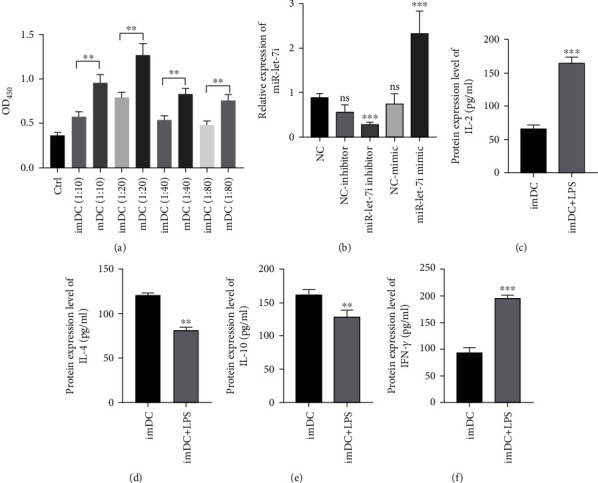
The effect of DC maturity on T cell biology. (a) CCK-8 detection of the T cell proliferation status of each group of mixed lymphatic systems. (b) RT–qPCR detection of the effect of each group of transfectants on miRNA-let-7i. (c–f) ELISA detection of IL-2 and IL-4 in each group. The production of IL-10 and IFN-*γ*. Each experiment was repeated at least 3 times, ^∗^*P* < 0.05, ^∗∗^*P* < 0.01, ^∗∗∗^*P* < 0.001, and ^∗∗∗∗^*P* < 0.0001.

ImDCs and mDCs (imDCs induced by LPS to maturation) were cultured with the extracted T cells. The CCK-8 results showed that at the same ratio, the proliferation of mDC-stimulated T cells was faster than that of imDC-stimulated T cells, and the ratio reached 1 : 20. T cell proliferation was the fastest (Figure 2(a)). The RT–qPCR results showed that after treating each group of cells with different miRNA-let-7i transfectants, they inhibited miRNA-let-7i and promoted miRNA-let. The ELISA results showed that the coculture of imDCs and T cells induced by LPS promoted the production of IL-2 and IFN-*γ* and inhibited the production of IL-4 and IL-10 (Figures 2(c)–2(f)). The above results indicate that the maturity state of DCs can affect the immune response of T cells.

### 3.3. Upregulation of miR-let-7i Promotes LPS-Induced DC Maturation

**Figure 3:**
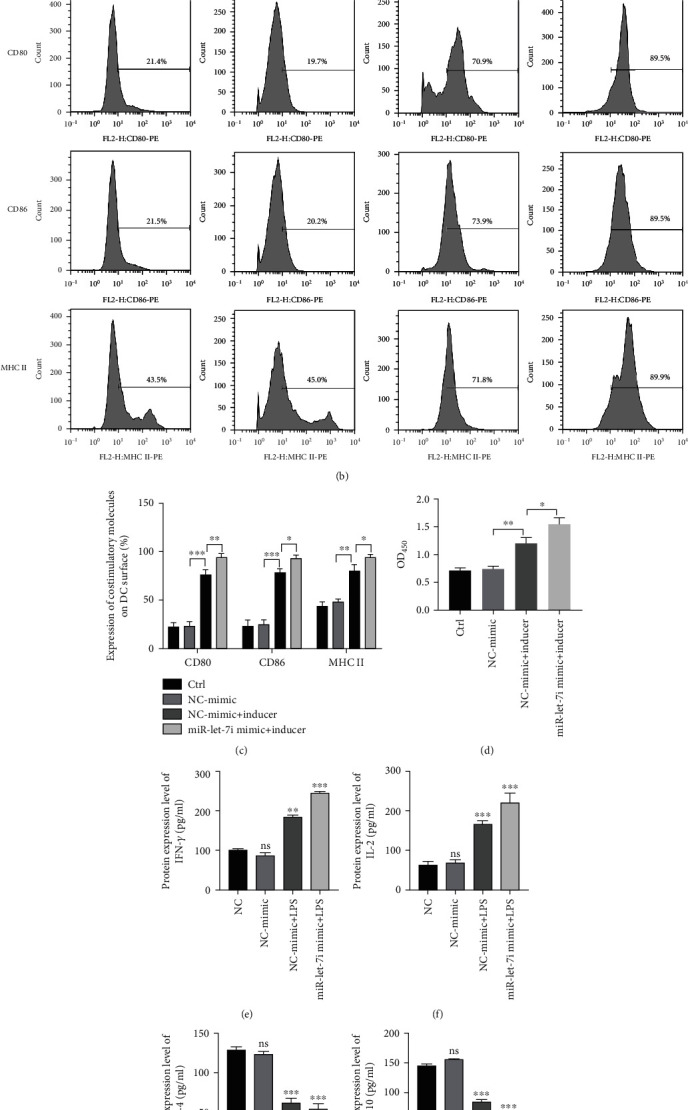
The miR-let-7i mimic promotes the maturation of imDCs. (a) Transmission electron microscopy to detect the morphological differences in DCs. (b and c) Flow cytometry to detect CD80, CD86, and MHC II expressions. (d) CCK-8 to detect T cell proliferation. (e–h) ELISA to detect IL-2 and the production of IL-4, IL-10, and IFN-*γ*. Each experiment was repeated at least 3 times, ^∗^*P* < 0.05, ^∗∗^*P* < 0.01, ^∗∗∗^*P* < 0.001, and ^∗∗∗∗^*P* < 0.0001.

The miR-let-7i mimic was transfected into imDCs, which were then treated with LPS and cultured with the extracted T cells. The transmission electron microscopy results showed that the miR-let-7i mimic promoted LPS-induced imDCs to the mature stage (Figure 3(a)). The flow cytometry results showed that the miR-let-7i mimic promoted the expression of the surface markers CD80, CD86, and MHC II (Figures 3(b) and 3(c)). The CCK-8 results showed that the miR-let-7i mimic promoted the proliferation of T cells in a mixed lymphatic system (Figure 3(d)). The ELISA results showed that the miR-let-7i mimic promoted the production of IL-2 and IFN-*γ* and inhibited the production of IL-4 and IL-10 (Figures 3(e)–3(h)). The above results indicate that the miR-let-7i mimic promotes the maturation of imDCs and affects the immune response of T cells, increasing immune rejection.

### 3.4. Downregulation of miR-let-7i Inhibits T Cell Responses Induced by Mature DCs

**Figure 4:**
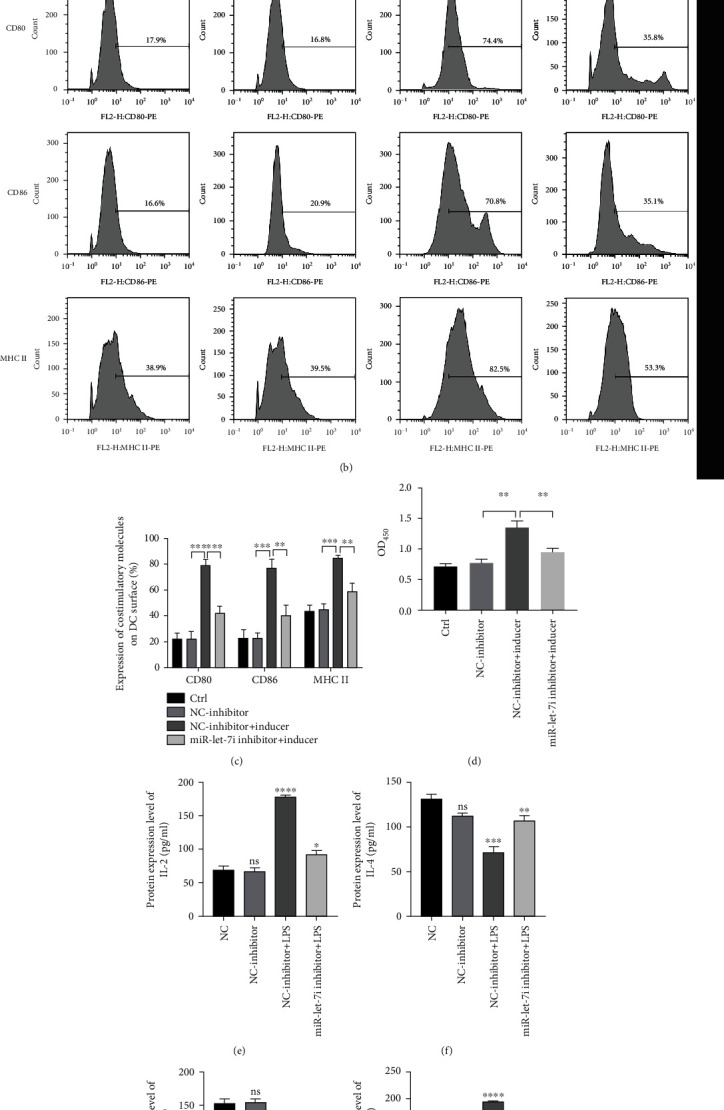
The miR-let-7i inhibitor maintains the immature state of imDCs. (a) Transmission electron microscopy to detect the morphological differences in DCs. (b and c) Flow cytometry to detect CD80, CD86, and MHC II expressions. (d) CCK-8 to detect T cell proliferation. (e–h) ELISA to detect IL-2 and the production of IL-4, IL-10, and IFN-*γ*. Each experiment was repeated at least 3 times, ^∗^*P* < 0.05, ^∗∗^*P* < 0.01, ^∗∗∗^*P* < 0.001, and ^∗∗∗∗^*P* < 0.0001.

The miR-let-7i inhibitor was transfected into imDCs, which were then treated with LPS and cultured with the extracted T cells. The transmission electron microscopy results showed that the miR-let-7i inhibitor inhibited LPS-induced imDCs to the mature stage (Figure 4(a)). The flow cytometry results showed that the miR-let-7i inhibitor inhibited the expression of the surface markers CD80, CD86, and MHC II (Figures 4(b) and 4(c)). The CCK-8 results showed that the miR-let-7i inhibitor inhibited the proliferation of T cells in the mixed lymphatic system (Figure 4(d)). The ELISA results showed that the miR-let-7i inhibitor inhibited the production of IL-2 and IFN-*γ* and promoted the production of IL-4 and IL-10 (Figures 4(e)–4(h)). The above results indicate that the miR-let-7i inhibitor inhibits the maturation of imDCs and affects the immune response of T cells, reducing immune rejection.

### 3.5. Downregulation of miR-let-7i Inhibits Skin Graft Rejection in Rhesus Monkeys

**Figure 5:**
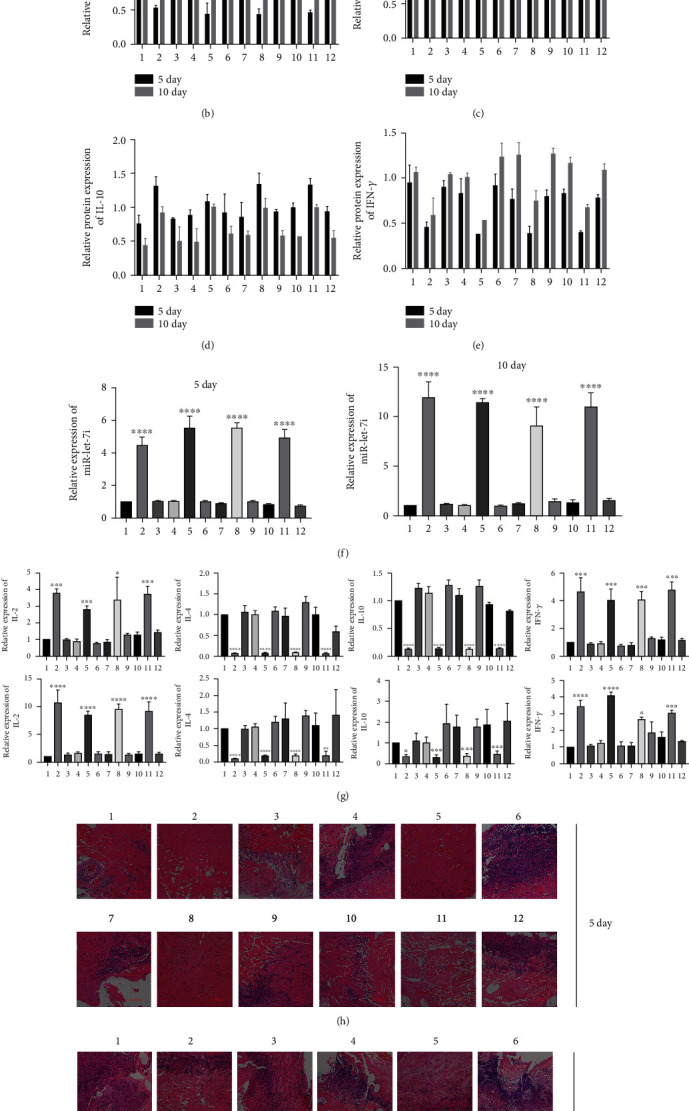
ImDCs transfected with the miR-let-7i inhibitor inhibited allograft rejection. (a–e) Western blot experiment to detect the production of IL-2, IL-4, IL-10, and IFN-*γ*. (f) RT–qPCR to detect the expression of miR-let-7i. (g) RT–qPCR to detect the expression of IL-2, IL-4, IL-10, and IFN-*γ* (upper: 5 days and lower: 10 days). (h and i) HE detects the condition of transplanted skin tissue. Each experiment was repeated at least 3 times for each sample, and 1-12 indicates the transplant site number, ^∗^*P* < 0.05, ^∗∗^*P* < 0.01, ^∗∗∗^*P* < 0.001, and ^∗∗∗∗^*P* < 0.0001.

The operation was performed in accordance with a previous method. One-quarter of each grafted skin graft was removed on the 5th day after transplantation, and the remaining 1/2 of the grafted skin graft was removed on the 10th day after transplantation for testing. The results of Western blot experiments showed that imDCs transfected with miR-let-7i inhibitor into the skin of rhesus monkeys can effectively inhibit the production of IL-2 and IFN-*γ*, and the inhibitory effect diminished over time (Figures 5(a)–5(e)). RT–qPCR results showed that imDCs transfected with miR-let-7i inhibitor could not effectively inhibit the expression of miR-let-7i in the transplanted skin under the skin. The expression was higher at 5 days (Figure 5(f)); ELISA results showed that IL-2 and IFN-*γ* were expressed at high levels at 5 and 10 days and that IL-4 and IL-10 were expressed at low levels (Figure 5(g)). The results of HE staining showed that imDC cells transfected with miR-let-7i inhibitor could effectively prolong the survival time of transplanted skin (Figures 5(h) and 5(i)). The pathological grading results showed that the miR-let-7i inhibitor group significantly reduced the rejection grade of grafted skin grafts 5 days and 10 days after transplantation compared with NC (Table 3). The skin pathological rejection grading refers to the composite tissue allograft pathological rejection grading standard of the Banff grading system [17]. The final results showed that downregulation of miR-let-7i expression could significantly inhibit skin graft rejection in rhesus monkeys, and its effect on acute rejection was greater than that in chronic rejection.

## 4. Discussion

Organ transplantation is one of the most important medical achievements in the 20th century. It is not only an effective treatment for patients with end-stage organ failure but can also improve their quality of life [18, 19]. Skin transplantation can save many patients with severe skin burns [20]. In the past few years, although the short-term clinical results of immunosuppressive therapy after transplantation have improved, it has been found that the long-term use of immunosuppressants can lead to severe infections, cardiovascular disease, renal insufficiency, and new malignant tumors. The life expectancy of patients after transplantation is reduced, which increases the incidence of psychological problems among patients and adversely affects their quality of life. The cost of long-term immunosuppressive drugs also places a very high economic burden on transplant patients and the medical system [4, 21, 22]. Therefore, finding other methods to promote immune tolerance is of great significance to improve the success rate of transplantation.

The initial trauma and tissue damage caused by allograft implantation are related to the initiation of the inflammatory process and the innate immune response. In this process, donor DCs migrate from the graft to secondary lymphoid organs of the recipient, present antigen to T cells (including helper T cells and cytotoxic T cells), and activate T cells (and produce interleukin 12) to promote T cell proliferation, triggering an antigen-specific immune response to destroy target cells [23–26]. In the body, the function of DCs is mainly related to their mature phenotype. mDCs produce CD40, CD80, CD86, and other costimulatory molecules to stimulate T cells to secrete a large amount of IL-2 and induce IFN-*γ* and other cytokines. Production stimulates the proliferation of T cells and chemoattracts them into Th1 cells at the same time and promotes immune rejection [15, 27]. ImDCs can express fewer costimulatory molecules. When imDCs are in contact with T cells, they secrete higher levels of IL-4, IL-10, and other cytokines, which can inhibit the secretion of IFN-*γ* and spur the differentiation of helper T cells into Th2 cells, inhibit Th1-driven rejection, and promote the formation of peripheral immune tolerance. In clinical organ transplantation, when combined with bone marrow cells containing imDC precursor cells, the survival time of the transplant can be significantly prolonged [28]. In this study, after imDCs and mDCs were mixed with T cells, the results showed that mature DCs significantly promoted the proliferation of T cells. At the same time, we found that after inhibiting the maturation and progression of DCs, the production levels of CD40, CD80, CD86, and other costimulatory molecules were significantly reduced and could effectively inhibit the production of IL-2 and IFN-*γ* and promote the production of IL-4 and IL-10, indicating that the maturity state of DCs can affect the immune response of T cells. These findings are consistent with previous research [29].

The maturity status of DCs may be a key factor that influences the induction of T cell tolerance by promoting the differentiation of regulatory T cells [30, 31]. Mature DCs can enhance T cell immunity, but immature DCs participate in the induction of T cell tolerance in peripheral blood. Therefore, the functional status of DCs depends to a certain extent on the maturity of the DCs. The transcriptional and translational regulation of miRNAs provides a new mechanism to fine-tune DC function and the immune response. Studies have shown that miRNA-26a prolongs the survival of mouse allogeneic skin transplantation and promotes the expansion of regulatory T cells [31]. The expression of miR-let-7i is upregulated in the maturation of DCs induced by lipopolysaccharide (LPS). Inhibition of let-7i can inhibit the maturation of DCs, induce T cell hyporeactivity, increase the proportion of Treg cells in vitro, and then induce cardiac allograft tolerance in a rat heart transplantation model [32]. In this study, we obtained similar results: let-7i expression was upregulated during the DC maturation process induced by LPS. To elucidate the effect of miR-let-7i expression on DC maturation and function under LPS stimulation, we examined whether changes in the basal level of miR-let-7i affected DC maturation and function under LPS stimulation. The experimental results showed that inhibiting the expression of miR-let-7i significantly inhibited the maturation of DCs and affected their function, which was manifested in the induction of low T cell reactivity and the inhibition of T cell proliferation. We also established a rhesus monkey skin transplantation model, subcutaneously injected imDCs transfected with empty vector and transfected imDCs with miR-let-7i gene inhibition and PBS solution derived from the induced and cultured peripheral blood of monkeys, and carried out skin transplantations. The skin grafts were removed and tested, and it was found that the expression levels of the inflammatory factors IL-2 and IFN-*γ* in the imDC group transfected with the miR-let-7i gene were significantly decreased, while the expression levels of IL-10 and IL-4 were significantly increased. These findings indicate that when the expression of miR-let-7i in the skin is inhibited, it can promote the immunosuppressive effect of CD4+CD25+ Tregs, revealing a new role of miR-let-7i in the immune tolerance of skin grafts.

In conclusion, our data prove the immunomodulatory effect of miRNA let-7i in skin transplantation. We showed that inhibiting miRNA let-7i can prolong the survival time of allogeneic skin transplantation, and by inhibiting the expression of IFN-*γ*, IL-2, and other inflammatory factors and promoting the expression of IL-4 and IL-10, helper T cells were able to differentiate into Th2 cells that inhibit Th1-driven rejection, which has also been confirmed in homologous skin transplantation experiments in rhesus monkeys. Although further research is needed to fully elucidate the precise molecular and cellular mechanisms involved in immune regulation, miRNA let-7i treatment may be a new therapeutic strategy to protect allogeneic skin grafts.

## Figures and Tables

**Table 1:** Basic characteristics of the experimental animals.

Serial number	Group number	Sex	Age (year)	Weight (kg)	Body length (cm)	Chest circumference (cm)	Waist circumference (cm)	Hip circumference (cm)
07071	A	Male	14	6.98	53.5	39.5	23.5	30.5
08045	B	Male	13	7.76	52.0	40.5	24.5	30.5
08355	C	Male	13	6.06	53.0	37.5	28.5	33.5
07333	D	Male	14	9.54	59.5	41.0	35.0	40.5

**Table 2:** Group classification of experimental animals.

Serial number	Group number	Blood type	Rh detection	Donor and recipient	Transplant site number	Injection cell number
07071	A	O	+ (positive)	07071 and 08045	1	08045-NC
					2	08045-inhi
					3	08045-PBS
08045	B	O	+ (positive)	07071 and 08045	4	07071-NC
					5	07071-inhi
					6	07071-PBS
08355	C	O	- (feminine)	08355 and 07333	7	07333-NC
					8	07333-inhi
					9	07333-PBS
07333	D	O	- (feminine)	08355 and 07333	10	08355-NC
					11	08355-inhi
					12	08355-PBS

**Table 3:** Pathological grading of allograft skin transplantation in rhesus monkey.

Serial number	Graft site	Pathology grade (5 days)	Pathology grade (10 days)
1	A-NC inhibitor	1 (B)	4 (E)
2	A-7i inhibitor	0 (A)	2 (C)
3	A-PBS	2 (C)	3 (D)
4	B-NC inhibitor	2 (C)	4 (E)
5	B-7i inhibitor	0 (A)	1 (B)
6	B-PBS	4 (E)	4 (E)
7	C-NC inhibitor	3 (D)	3 (D)
8	C-7i inhibitor	1 (B)	1 (B)
9	C-PBS	3 (D)	4 (E)
10	D-NC inhibitor	2 (C)	4 (E)
11	D-7i inhibitor	0 (A)	3 (D)
12	D-PBS	4 (E)	4 (E)

## Data Availability

The data used to support the research are included within this article.
